# Crystal structures of di-μ-chlorido-bis­({(*E*)-5-(ethyl­amino)-4-methyl-2-[(pyridin-2-yl)diazen­yl]phen­o­lato}copper(II)) and chlorido­bis­(1,10-phen­anthroline)copper(II) chloride tetra­hydrate

**DOI:** 10.1107/S205698902300138X

**Published:** 2023-02-21

**Authors:** Alex Meier, Mohammad Rasel Mian, Siyu Ou, Scott Lovell, Minae Mure

**Affiliations:** a Department of Chemistry, The University of Kansas, Lawrence, KS 66045, USA; b X-ray Crystallography Laboratory, The University of Kansas, Lawrence, KS 66045, USA; c Protein Structure Laboratory, The University of Kansas, Lawrence, KS66047, USA; University of Durham, United Kingdom

**Keywords:** X-ray crystallography, copper(II) complex, chlorido bridge, supra­molecular features

## Abstract

The dark-red title complex crystallized from an equimolar methanol solution of (*E*)-5-(ethyl­amino)-4-methyl-2-[(pyridin-2-yl)diazen­yl]phenol and CuCl_2_(phen) (phen = 1,10-phenanthroline) as a centrosymmetric dimer, [CuCl(C_14_H_15_N_4_O)]_2_. The Cu atoms are bridged by two Cl ligands and have a slightly distorted square-pyramidal coordination, where two N atoms from the azo and the pyridine moieties, a phenolic O and a Cl atom comprise the base and the other Cl occupies the apex position. The apical Cu—Cl bond, 2.6192 (4) Å, is longer than the basal one, 2.2985 (3) Å, due to Jahn–Teller distortion. The dimers are associated *via* weak inter­molecular hydrogen bonds and π–π stacking inter­actions between phenyl and pyridine rings. A monomeric by-product of the same reaction, [CuCl(phen)_2_]Cl·4H_2_O, has a trigonal–bipyramidal coordination of Cu with equatorial Cl ligand, and extensive outer-sphere disorder. In the structure of **4**, the packing of cations leaves continuous channels containing disordered Cl^−^ anions and solvent mol­ecules. The identity of the solvent (water or a water/methanol mixture) was not certain. The disordered anion/solvent regions comprise 28% of the unit-cell volume. The disorder was approximated by five partly occupied positions of the Cl^−^ anion and ten positions of O atoms with a total occupancy of 3, giving a total of 48 electrons per asymmetric unit, in agreement with the integral electron density of 47.8 electrons in the disordered region, as was estimated using the BYPASS-type solvent-masking program [van der Sluis & Spek (1990). *Acta Cryst.* A**46**, 194–201].

## Abstract 2

(*E*)-5-(Ethyl­amino)-4-methyl-2-[(pyridin-2-yl)diazen­yl]phenol forms a centrosymmetric dimeric Cu^II^ complex with a double apical-basal chlorido bridge between square-pyramidal Cu centers, while 1,10-phenanthroline forms a monomeric trigonal–bipyramidal cation with equatorial Cl ligand.

## 1. Chemical context

**Figure 1:**
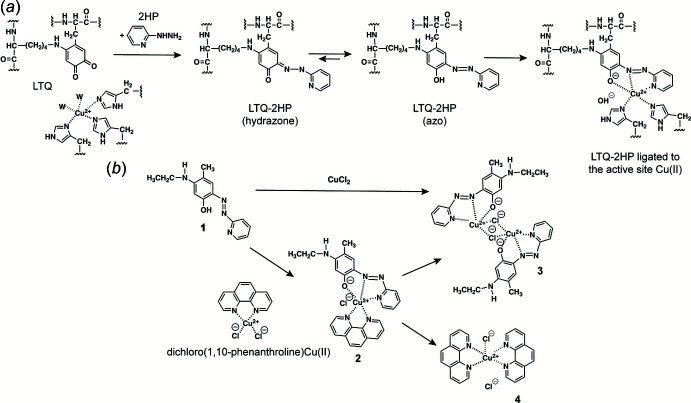
(*a*) The covalent modification of the LTQ cofactor of LOXL2 by 2HP. After the tautomerization of the hydrazone to the azo form, LTQ-2HP ligates to the active site Cu^2+^. The 2HP-modified LTQ (LTQ-2HP) containing the peptide was detected by mass spectrometry (Meier, Go, *et al.*, 2022 ▸). Based on the close resemblances of UV–vis and resonance Raman spectra of 2HP-inhibited LOXL2 and the model compound **2**, we hypothesize that LTQ-2HP serves as a tridentate ligand to the active site Cu^II^ in LOXL2 (Meier, Moon *et al.*, 2022 ▸). The +2 charge of Cu^II^ is expected to be canceled out by the 4-oxoanion of LTQ-2HP and a nearby acidic residue or a water mol­ecule (Meier, Kuczera *et al.*, 2022 ▸). (*b*) During the recrystallization of the dark red solids (**2**) isolated from an equimolar mixture of the LTQ-2HP model compound (**1**) and CuCl_2_(phen) in anhydrous methanol, we first isolated dark-red crystals (**3**), then also isolated (**4**) from the mother liquor that was left for a week at room temperature.

**Figure 2:**
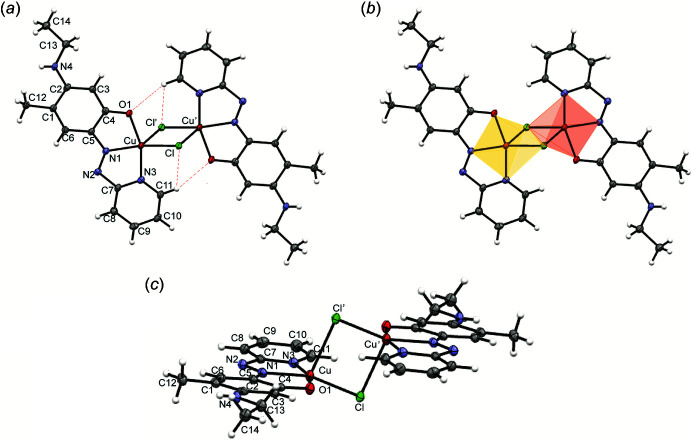
Mol­ecular structure of **3** in different aspects (*a*, *c*), showing the coordination polyhedra of Cu (*b*) and intra­molecular hydrogen bonds (*a*). Atomic displacement ellipsoids are drawn at the 50% probability level. Primed atoms are generated by inversion, symmetry operation 1 − *x*, 1 − *y*, −*z*.

The (*E*)-5-(ethyl­amino)-4-methyl-2-[(pyridin-2-yl)diazen­yl]phenol ligand (**1**) was synthesized from a coupling reaction of pyridine-2-diazo­tate and 3-ethyl­amino-*p*-cresol as a model for the lysine tyrosyl­quinone (LTQ) cofactor (Fig. 1 ▸) of lysyl oxidase-like **2** (LOXL2) that is inhibited by 2-hydrazino­pyridine (2HP). LOXL2 is a member of the lysyl oxidase family of proteins, and its upregulation has been closely associated with fibrosis and tumor metastasis (Moon *et al.* 2014 ▸; Mahjour *et al.*, 2019 ▸; Wei *et al.*, 2021 ▸). We have recently identified 2HP-modified LTQ, LTQ-2HP (Fig. 1 ▸) in 2HP-inhibited LOXL2 by mass spectrometry-based peptide mapping (Meier, Go *et al.*, 2022 ▸). Since there is no structural information of a catalytically competent form of LOXL2, we conducted comparative spectroscopic studies of 2HP-inhibited LOXL2 and the corresponding model compound in solution, in order to understand the spatial arrangement of the LTQ cofactor and the active site Cu^II^ (Meier, Moon *et al.*, 2022 ▸). The UV–vis spectroscopic feature of 2HP-inhibited LOXL2 indicated the ligation of LTQ-2HP to the active site Cu^II^ (Fig. 2 ▸). 
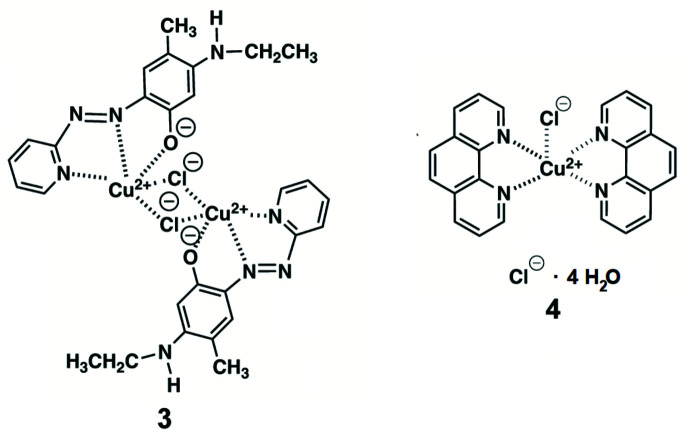


In order to model the LTQ-2HP ligated to the active site Cu^II^, **1** was mixed with an equimolar amount of di­chloro(phen)Cu (phen = 1,10-phenanthroline) in anhydrous methanol to isolate dark-red solids (**2**), where the phen ligand was used to mimic two of the three His ligands of the active site Cu^II^ in LOXL2 (Meier, Kuczera *et al.*, 2022 ▸). Upon slow evaporation of methanol solution of **2**, dark-red crystals (**3**) were isolated and characterized as a dimeric complex [CuCl(C_14_H_15_N_4_O)]_2_ (Fig. 1 ▸).

After isolation of **3**, green prismatic crystals (**4**) were isolated from the mother liquor and identified as a monomeric complex, [CuCl(phen)_2_]^−^ Cl^+^·4H_2_O (Fig. 1 ▸). Herein we report the crystal structures of **3** and **4**.

## 2. Structural commentary

The mol­ecule of **3** (Fig. 2 ▸) has a crystallographic inversion center. Each Cu atom is penta-coordinated by N1, N3, and the deprotonated O1 of the oxoanion **1**, as well as two inversion-related bridging chloride ligands, Cl and Cl’. Atoms N1, N3, O1 and Cl are nearly coplanar and comprise the base of a distorted square pyramid while Cl’ occupies the apical position. The apical Cu—Cl bond is *ca* 0.32 Å longer than the basal one due to the Jahn–Teller effect (Addison *et al.*, 1984 ▸). The Addison parameter, τ = (β – α)/60° = 0.007 (where α = 160.67° and β = 161.00° are the widest bond angles) indicates a small distortion from an ideal square-pyramidal geometry (τ = 0) towards a trigonal–bipyramidal geometry (τ = 1). The coord­ination polyhedra of the two Cu atoms share one base-to-apex edge (Fig. 1 ▸ *b*), while their basal planes are rigorously parallel to each other (with an inter­planar separation of 1.789 Å), in a type II arrangement as classified by Rodriguez *et al.* (1999 ▸). The Cu_2_Cl_2_ plane is perpendicular to the basal planes. The geometry agrees with that in other Cu_2_(μ-Cl)_2_ centers (Sasmal *et al.*, 2013 ▸; Rodriguez *et al.*, 1999 ▸). In the ligand **1**, the aromatic phenyl and pyridine rings are conjugated through the N=N (azo) bond of 1.301 (2) Å and adopt a *E*, or *trans*, configuration about this bond, with a C—N=N—C torsion angle of −179.0 (1)°. The dimer also contains two pairs of weak intra­molecular hydrogen bonds, C11—H11⋯Cl and C11—H11⋯O1 (Table 1 ▸).

**Figure 3:**
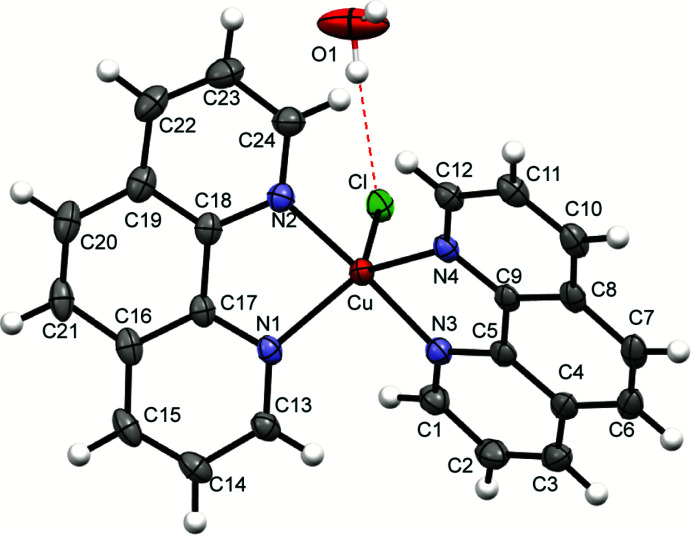
The cation and ordered water mol­ecule in the structure of **4**. Atomic displacement ellipsoids are drawn at the 50% probability level.

The asymmetric unit in the structure of **4** contains one monomeric cation (Fig. 3 ▸) in which the Cu^II^ atom has a distorted trigonal–bipyramidal coordination (τ = 0.848) with two chelating 1,10-phenanthroline ligands and one Cl atom, the latter in an equatorial position. A similar coordination geometry was observed in monomeric Cu^II^ complexes [Cu(CN)(phen)_2_]NO_3_ (Anderson, 1974) and [CuCl(5,6-di­methyl-1,10-phenanthroline)_2_]PF_6_ (Yamada, 2002 ▸), although the Cu—Cl bond in the latter [2.257 (1) Å] is much shorter than in **4** [2.3527 (6) Å].

## 3. Supra­molecular features

**Figure 4:**
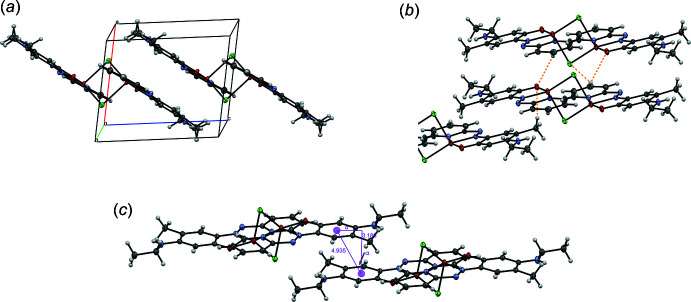
Crystal packing of **3** (*a*), showing inter­molecular hydrogen bonds (*b*) and phen­yl–phenyl π–π stacking inter­actions (*c*) (α is the angle between the ring normal and centroid–centroid vector, *d* is the displacement between the rings).

The crystal packing of **3** is shown in Fig. 4 ▸. Each mol­ecule forms ten weak inter­molecular hydrogen bonds C—H⋯*X*, where *X* = Cl or O (Grabowski, 2021 ▸). The Cl atom is engaged in four such inter­actions and the O atom in two (supporting Fig. 1A). Additional stabilization is provided by off-center parallel π–π stacking inter­actions (Janiak, 2000 ▸; Martinez & Iverson, 2012 ▸) between two phenyl rings, between two pyridine rings, or between a phenyl and a pyridine ring (Fig. 4 ▸ and supporting Fig. 1B,C). The distances between ring centers (centroid–centroid distances), the distances between the ring center and the plane of the ring (plane-plane distances) and the α angle between the ring normal and the center of the opposite ring of the three modes of π–π inter­actions are summarized in Table 2 ▸. Remarkably, the amino-H atom is not engaged in any hydrogen bond, probably due to screening by two adjacent methyl groups.

In the structure of **4** (supplemental Fig. 2), the packing of cations leaves continuous channels containing disordered Cl^−^ anions and solvent mol­ecules. Of the latter, one water mol­ecule per asymmetric unit is ordered, being ‘anchored’ by an O1—H1*A*⋯Cl1 hydrogen bond with the cation [O1⋯Cl1 = 3.173 (3), H1*A*⋯Cl1 = 2.34 Å]. The rest of the solvent is intensely disordered and its identity (water or a water/methanol mixture) was not certain. The disordered anion/solvent regions comprise 28% of the unit-cell volume. The disorder was approximated by five partly occupied positions of the Cl^−^ anion and ten positions of O atoms with a total occupancy of 3 – presumably water mol­ecules whose hydrogen atoms could not be located. This gives a total of 48 electrons per asymmetric unit, in agreement with the integral electron density of 47.8 electrons in the disordered region, as was estimated using the BYPASS-type solvent-masking program (van der Sluis & Spek, 1990 ▸) on the *OLEX2* platform (Dolomanov *et al.*, 2009 ▸).

## 4. Database survey

Several crystal structures of penta-coordinated centrosymmetric Cu^II^ dimers with the Cu atoms bridged by two Cl ligands and bonded to ligands with N and O atoms, have been deposited in the Cambridge Structural Database (CSD, Version 5.38; Groom *et al.*, 2016 ▸), *viz*. FEWFAO (Rodriguez *et al.*, 1999 ▸), MUNWIB, MUNWOH (Kapoor *et al.*, 2002 ▸), YECGUK (Das *et al.*, 2012 ▸), SIDQED (Sasmal *et al.*, 2013 ▸), and POJKOQ (Smolentsev *et al.*, 2014 ▸). However, no complexes with ligand **1** were found. To our knowledge, **3** is the first example of a penta-coordinated centrosymmetric Cu^II^ dimer in which the Cu atoms are bridged by two Cl ligands and are bonded each to two N atoms (pyridine N and aromatic –N=N–) and a phen­oxy-O atom. There are multiple structures of phen and its derivatives complexed with Cu^II^, the two structures closely related to **4** being PENCUN (Anderson, 1975 ▸) and XUMZOU (Yamada *et al.*, 2002 ▸), see Section 2.

## 5. Synthesis and crystallization

### 5.1. Synthesis of pyridine-2-diazo­tate

Isoamyl nitrite (4.03 ml, 30 mmol) was added to a slurry of 2-amino­pyridine (2.82 g, 30 mmol) and sodium amide (1.29 g, 33 mmol) in 30 ml of anhydrous THF and the reaction mixture was refluxed for 2 h (Bunton *et al.*, 1974 ▸). After cooling to room temperature, precipitates were isolated by vacuum filtration, washed with tetra­hydro­furan (THF) and dried under vacuum. Pyridine-2-diazo­ate was isolated as a pale-yellow solid (2 g, 63%) ^1^H NMR (400 MHz, DMSO-*d*_6_) δ 8.26 (*d*, *J* = 3.7 Hz, 1H), 7.55 (*dd*, *J* = 7.7 Hz, 1H), 7.39 (*d*, *J* = 8.2 Hz, 1H), 6.91 (*dd*, *J* = 7.7 Hz, 1H).

### 5.2. Synthesis of (*E*)-5-(ethyl­amino)-4-methyl-2-[(pyridin-2-yl)diazen­yl]phenol, **1**

3-Ethyl­amino-*p*-cresol (5.3 g, 35.1 mmol) was added to the suspension of pyridine-2-diazo­tate (7.5 g, 70.8 mmol) in 100 ml of ethanol and the pH of the reaction mixture was adjusted to 8 by aqueous HCl (Nakagawa & Wada, 1962 ▸). After refluxing for 2 h, the solvent was removed under reduced pressure. The resulting solids were washed with water and dried *in vacuo*. (*E*)-5-(Ethyl­amino)-4-methyl-2-[(pyridin-2-yl)diazen­yl]phenol, **1**, was isolated as a dark-red solid (4.59 g, 51%). ^1^H NMR (400 MHz, chloro­form-*d*) δ 16.05 (*s*, 1H), 8.40 (*d*, *J* = 4.1 Hz, 2H), 7.75–7.66 (*m*, 2H), 7.55 (*d*, *J* = 8.3 Hz, 2H), 7.06–6.98 (*m*, 2H), 6.92 (*s*, 2H), 5.74 (*s*, 2H), 4.63 (*s*, 1H), 3.28 (*dt*, *J* = 13.4, 7.2 Hz, 4H), 2.10 (*s*, 5H), 1.33 (*t*, *J* = 7.2 Hz, 6H). ^13^C NMR (101 MHz, chloro­form-*d*) δ 175.67, 156.20, 155.56, 148.78, 138.06, 133.91, 133.11, 121.71, 119.72, 110.42, 97.71, 38.17, 16.51, 14.08. HRMS (ESI+) C_14_H_18_N_4_O (*M* ^+^ + 1) calculated: 257.1402, observed 257.1419.

### 5.3. Crystallization

**Figure 5:**
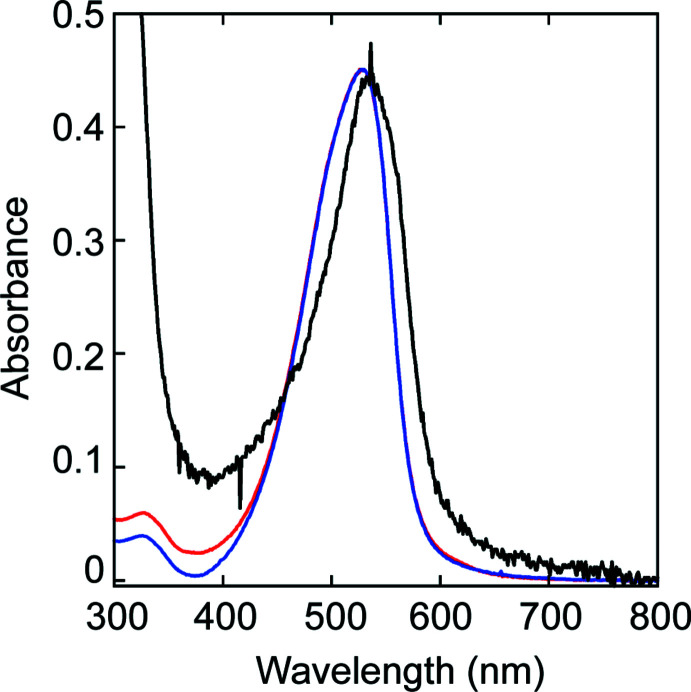
UV–vis spectra of 2HP-inhibited LOXL2 (LOXL2–2HP) (in black) (Meier, Moon *et al.*, 2022 ▸), crystalline **3** isolated from a 1:1 mixture of **2** with CuCl_2_(phen) (in blue), and crystalline **3** isolated from a 1:1 mixture of **2** with CuCl_2_ (in red). All spectra were recorded in 50 m*M* HEPBS buffer (pH 8.0).

Compound **1** was purified by recrystallization from methanol by slow evaporation. Dark-yellow needle-like crystals of **1** were obtained after a week at room temperature. CuCl_2_(phen) (123 mg, 0.39 mmol) was added to a suspension of **1** (100 mg, 0.39 mmol) in 5 ml of methanol. The reaction mixture was sonicated to completely dissolve solids and subjected to slow evaporation of methanol at room temperature. Dark-red single crystals of **3**, suitable for X-ray crystallography, were obtained within a day. After removing the crystals of **3**, small green crystals of **4** were formed from the mother liquor. Recrystallization of **3** by slow evaporation of an equimolar mixture of **1** in methanol and CuCl_2_ in a minimal amount of water at room temperature gave dark-red crystals within a couple of days (Fig. 1 ▸). The UV–vis spectra of crystalline **3** obtained by two methods are identical and superimposable to the visible region of the UV–vis spectrum of 2HP-inhibited LOXL2 (Fig. 5 ▸). These results strongly support our hypothesis that 2HP-inhibited LOXL2 contains LTQ-2HP that is ligated to the active site Cu^2+^ and the LTQ cofactor resides in the vicinity of the Cu^2+^ center (Meier, Moon *et al.*, 2022 ▸; Meier, Kuczera *et al.*, 2022 ▸).

## 6. Refinement

Crystal data, data collection and structure refinement details are summarized in Table 3 ▸. In **3**, all H atoms were refined in isotropic approximation. In **4**, the H atoms of the disordered water mol­ecules were ignored, H1*A* was refined in an isotropic approximation, other H atoms were placed in idealized positions (C—H = 0.95, O—H = 0.84 Å) and refined as riding on their carrier atoms with *U*_iso_(H) = 1.2*U*_eq_(C) or 1.5*U*_eq_(O). The treatment of the disorder is described in the *Supra­molecular features* section.

## Supplementary Material

Crystal structure: contains datablock(s) global, 3, 4. DOI: 10.1107/S205698902300138X/zv2019sup1.cif

Structure factors: contains datablock(s) 3. DOI: 10.1107/S205698902300138X/zv20193sup2.hkl

Structure factors: contains datablock(s) 4. DOI: 10.1107/S205698902300138X/zv20194sup3.hkl

Supplementary figures. DOI: 10.1107/S205698902300138X/zv2019sup4.docx

CCDC references: 2219280, 2219281

Additional supporting information:  crystallographic information; 3D view; checkCIF report

## Figures and Tables

**Table 1:** Hydrogen bonds (Å, °) in the crystal of **3**

*D*—H⋯*A*	*d*(*D*—H)	*d*(H⋯*A*)	*d*(*D*⋯*A*)	<(*DHA*)
Intra­molecular				
C11—H11⋯O1^i^	0.92 (2)	2.60 (2)	3.4083 (18)	146.5 (19)
C11—H11⋯Cl*^*a*^*	0.92 (2)	2.93 (2)	3.4825 (15)	120.2 (17)
Inter­molecular				
C9—H9⋯O1^ii^	0.92 (3)	2.59 (3)	3.1592 (18)	120.2 (17)
C9—H9⋯Cl^ii,a^	0.92 (3)	2.91 (3)	3.6514 (14)	138 (2)
C12—H12*B*⋯O1^iii^	0.96 (3)	2.85 (3)	3.4542 (15)	111.9 (17)
C12—H12*C*⋯Cl^iv^,*^*a*^*	0.93 (2)	3.00 (2)	3.790 (2)	169 (2)
C14—H14*B*⋯Cl^v^,*^*a*^*	0.97 (3)	2.94 (3)	3.6821 (16)	134.8 (19)

Symmetry transformations used to generate equivalent atoms: (i) −*x* + 1, −*y*, −*z*; (ii) *x*, *y* + 1, *z*; (iii) −*x*, 1 − *y*, 1 − *z*; (iv) 1 − *x*, 1 − *y*, 1 − *z*; (v) *x* − 1, *y*, *z* + 1. Note: (*a*) Very weak, if any, at the borderline of a hydrogen bond (Grabowski, 2021 ▸).

**Table 2:** Distances and α angle (Å, °) of inter­molecular π–π inter­actions in **3**

	Phen­yl–phen­yl	phen­yl–pyridine	pyridine–pyridine
Centroid–centroid distance	3.910 (1)	4.266 (1)	4.220 (1)
Plane–plane distance	3.433 (1)	3.534 (1)	3.499 (1)
α	28.60	34.06	33.99

**Table 3:** Experimental details

	**3**	**4**
Crystal data		
Chemical formula	[Cu_2_Cl_2_(C_14_H_15_N_4_O)_2_]	[CuCl(C_12_H_8_N_2_)_2_]Cl·4H_2_O
*M*_r_	708.58	566.91
Crystal system, space group	Triclinic, *P* 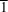	Monoclinic, *C*2/*c*
Temperature (K)	100	100
*a*, *b*, *c* (Å)	8.6965 (3), 8.7974 (4), 9.5574 (4)	23.1874 (7), 30.2708 (9), 7.2839 (2)
α, β, γ (°)	88.6165 (17), 79.3644 (16), 73.0017 (15)	90, 97.235 (1), 90
*V* (Å^3^)	686.90 (5)	5071.9 (3)
*Z*	1	8
Radiation type	Mo *K*α	Mo *K*α
μ (mm^−1^)	1.79	1.11
Crystal size (mm)	0.1 × 0.05 × 0.02	0.2 × 0.1 × 0.05

Data collection		
Diffractometer	Bruker D8 Venture	Bruker D8 Venture
Absorption correction	Multi-scan (*SADABS*; Krause et al., 2015 ▸)	Multi-scan (*SADABS*; Krause et al., 2015 ▸)
*T*_min_, *T*_max_	0.89, 0.94	0.89, 0.95
No. of measured, independent and observed [*I* > 2σ(*I*)] reflections	120912, 7366, 6202	67930, 6173, 5840
*R*_int_	0.047	0.029
(sin θ/λ)_max_ (Å^−1^)	0.862	0.667

Refinement		
*R*[*F* ^2^ > 2σ(*F* ^2^)], *wR*(*F* ^2^), *S*	0.035, 0.090, 1.03	0.045, 0.130, 1.05
No. of reflections	7366	6173
No. of parameters	250	364
H-atom treatment	All H-atom parameters refined	H atoms treated by a mixture of independent and constrained refinement
		
Δρ_max_, Δρ_min_ (e Å^−3^)	0.75, −0.87	1.06, −0.48

Computer programs: *APEX4* (Bruker, 2021 ▸), *SAINT* (Bruker, 2016 ▸), *SHELXT2014/5* (Sheldrick, 2015*a* ▸), *SHELXL2018/3* (Sheldrick, 2015*b* ▸), and *OLEX2* (Dolomanov *et al.*, 2009 ▸).

## supplementary crystallographic information

### Di-µ-chlorido-bis({(E)-5-(ethylamino)-4-methyl-2-[(pyridin-2-\ yl)diazenyl]phenolato}copper(II)) (3) Crystal data

**Table:**

[Cu_2_Cl_2_(C_14_H_15_N_4_O)_2_]	*Z* = 1
*M**_r_* = 708.58	*F*(000) = 362
Triclinic, *P*1	*D*_x_ = 1.713 Mg m^−^^3^
*a* = 8.6965 (3) Å	Mo *K*α radiation, λ = 0.71073 Å
*b* = 8.7974 (4) Å	Cell parameters from 9951 reflections
*c* = 9.5574 (4) Å	θ = 2.4–37.9°
α = 88.6165 (17)°	µ = 1.79 mm^−^^1^
β = 79.3644 (16)°	*T* = 100 K
γ = 73.0017 (15)°	Plate, clear dark red
*V* = 686.90 (5) Å^3^	0.1 × 0.05 × 0.02 mm

### Di-µ-chlorido-bis({(E)-5-(ethylamino)-4-methyl-2-[(pyridin-2-\ yl)diazenyl]phenolato}copper(II)) (3) Data collection

**Table:**

Bruker D8 Venture diffractometer	6202 reflections with *I* > 2σ(*I*)
φ and ω scans	*R*_int_ = 0.047
Absorption correction: multi-scan (SADABS; Krause et al., 2015)	θ_max_ = 37.8°, θ_min_ = 2.2°
*T*_min_ = 0.89, *T*_max_ = 0.94	*h* = −14→14
120912 measured reflections	*k* = −15→15
7366 independent reflections	*l* = −16→16

### Di-µ-chlorido-bis({(E)-5-(ethylamino)-4-methyl-2-[(pyridin-2-\ yl)diazenyl]phenolato}copper(II)) (3) Refinement

**Table:**

Refinement on *F*^2^	Primary atom site location: dual
Least-squares matrix: full	Secondary atom site location: difference Fourier map
*R*[*F*^2^ > 2σ(*F*^2^)] = 0.035	Hydrogen site location: difference Fourier map
*wR*(*F*^2^) = 0.090	All H-atom parameters refined
*S* = 1.02	*w* = 1/[σ^2^(*F*_o_^2^) + (0.036*P*)^2^ + 0.8401*P*] where *P* = (*F*_o_^2^ + 2*F*_c_^2^)/3
7366 reflections	(Δ/σ)_max_ < 0.001
250 parameters	Δρ_max_ = 0.75 e Å^−^^3^
0 restraints	Δρ_min_ = −0.87 e Å^−^^3^

### Di-µ-chlorido-bis({(E)-5-(ethylamino)-4-methyl-2-[(pyridin-2-\ yl)diazenyl]phenolato}copper(II)) (3) Special details

**Table:**

Geometry. All esds (except the esd in the dihedral angle between two l.s. planes) are estimated using the full covariance matrix. The cell esds are taken into account individually in the estimation of esds in distances, angles and torsion angles; correlations between esds in cell parameters are only used when they are defined by crystal symmetry. An approximate (isotropic) treatment of cell esds is used for estimating esds involving l.s. planes.

### Di-µ-chlorido-bis({(E)-5-(ethylamino)-4-methyl-2-[(pyridin-2-\ yl)diazenyl]phenolato}copper(II)) (3) Fractional atomic coordinates and isotropic or equivalent isotropic displacement parameters (Å^2^)

**Table:**

	*x*	*y*	*z*	*U*_iso_*/*U*_eq_	
Cu1	0.50615 (2)	0.52883 (2)	0.17788 (2)	0.01407 (4)	
Cl	0.69781 (4)	0.37524 (4)	−0.00149 (3)	0.01526 (6)	
O1	0.40583 (13)	0.36521 (12)	0.25883 (10)	0.01722 (17)	
N1	0.40360 (14)	0.63488 (13)	0.36379 (11)	0.01349 (17)	
N2	0.41967 (14)	0.77150 (13)	0.39617 (12)	0.01471 (18)	
N3	0.57379 (14)	0.72690 (14)	0.16097 (12)	0.01486 (18)	
N4	0.04781 (15)	0.28003 (15)	0.67389 (12)	0.01713 (19)	
H4	−0.004 (3)	0.314 (3)	0.747 (3)	0.029 (6)*	
C1	0.13310 (16)	0.51697 (16)	0.66217 (13)	0.01461 (19)	
C2	0.13569 (16)	0.36764 (15)	0.59774 (13)	0.01418 (19)	
C3	0.22722 (16)	0.31670 (16)	0.46195 (13)	0.0150 (2)	
H3	0.228 (3)	0.226 (3)	0.421 (3)	0.024 (6)*	
C4	0.32015 (16)	0.40792 (15)	0.38531 (13)	0.01372 (19)	
C5	0.31703 (15)	0.55528 (15)	0.45068 (13)	0.01324 (18)	
C6	0.22484 (16)	0.60569 (16)	0.58941 (13)	0.0148 (2)	
H6	0.228 (3)	0.703 (3)	0.629 (2)	0.017 (5)*	
C7	0.51507 (16)	0.82151 (15)	0.28082 (13)	0.01416 (19)	
C8	0.54550 (18)	0.96791 (16)	0.28910 (15)	0.0172 (2)	
H8	0.503 (3)	1.033 (3)	0.375 (3)	0.023 (6)*	
C9	0.63549 (18)	1.01756 (17)	0.17109 (16)	0.0191 (2)	
H9	0.653 (3)	1.115 (3)	0.180 (3)	0.032 (7)*	
C10	0.69318 (19)	0.92108 (18)	0.04851 (16)	0.0207 (2)	
H10	0.750 (3)	0.952 (3)	−0.035 (3)	0.032 (7)*	
C11	0.65869 (18)	0.77677 (17)	0.04711 (15)	0.0188 (2)	
H11	0.685 (3)	0.710 (3)	−0.032 (2)	0.021 (5)*	
C12	0.02602 (18)	0.57081 (19)	0.80418 (14)	0.0187 (2)	
H12A	0.060 (3)	0.489 (3)	0.876 (3)	0.027 (6)*	
H12B	−0.086 (3)	0.582 (3)	0.802 (3)	0.032 (6)*	
H12C	0.033 (3)	0.668 (3)	0.835 (3)	0.026 (6)*	
C13	0.03874 (19)	0.12852 (17)	0.62425 (15)	0.0186 (2)	
H13A	0.146 (3)	0.055 (3)	0.590 (2)	0.022 (5)*	
H13B	−0.020 (3)	0.149 (3)	0.549 (3)	0.022 (5)*	
C14	−0.0508 (2)	0.05186 (18)	0.74318 (16)	0.0210 (2)	
H14A	−0.056 (3)	−0.046 (3)	0.710 (2)	0.021 (5)*	
H14B	−0.160 (3)	0.118 (3)	0.781 (3)	0.031 (6)*	
H14C	0.007 (3)	0.025 (3)	0.821 (2)	0.015 (5)*	

### Di-µ-chlorido-bis({(E)-5-(ethylamino)-4-methyl-2-[(pyridin-2-\ yl)diazenyl]phenolato}copper(II)) (3) Atomic displacement parameters (Å^2^)

**Table:**

	*U*^11^	*U*^22^	*U*^33^	*U*^12^	*U*^13^	*U*^23^
Cu1	0.01659 (8)	0.01450 (7)	0.01084 (6)	−0.00724 (5)	0.00253 (5)	0.00021 (5)
Cl	0.01503 (12)	0.01630 (12)	0.01354 (11)	−0.00511 (9)	0.00058 (9)	−0.00025 (9)
O1	0.0209 (4)	0.0173 (4)	0.0123 (4)	−0.0080 (3)	0.0040 (3)	−0.0005 (3)
N1	0.0132 (4)	0.0147 (4)	0.0128 (4)	−0.0055 (3)	−0.0010 (3)	0.0019 (3)
N2	0.0164 (5)	0.0154 (4)	0.0126 (4)	−0.0071 (4)	0.0006 (3)	0.0004 (3)
N3	0.0157 (4)	0.0162 (4)	0.0124 (4)	−0.0066 (4)	0.0012 (3)	0.0014 (3)
N4	0.0190 (5)	0.0194 (5)	0.0134 (4)	−0.0094 (4)	0.0018 (4)	0.0022 (4)
C1	0.0137 (5)	0.0191 (5)	0.0106 (4)	−0.0059 (4)	0.0006 (4)	0.0014 (4)
C2	0.0136 (5)	0.0168 (5)	0.0122 (4)	−0.0057 (4)	−0.0009 (4)	0.0033 (4)
C3	0.0155 (5)	0.0164 (5)	0.0129 (4)	−0.0066 (4)	0.0011 (4)	0.0013 (4)
C4	0.0142 (5)	0.0148 (5)	0.0120 (4)	−0.0053 (4)	−0.0002 (4)	0.0020 (3)
C5	0.0132 (5)	0.0157 (5)	0.0109 (4)	−0.0060 (4)	0.0003 (3)	0.0019 (3)
C6	0.0152 (5)	0.0176 (5)	0.0116 (4)	−0.0064 (4)	0.0002 (4)	0.0012 (4)
C7	0.0145 (5)	0.0142 (5)	0.0132 (4)	−0.0050 (4)	0.0002 (4)	0.0009 (4)
C8	0.0196 (6)	0.0150 (5)	0.0171 (5)	−0.0070 (4)	−0.0004 (4)	0.0007 (4)
C9	0.0197 (6)	0.0162 (5)	0.0216 (6)	−0.0082 (4)	−0.0001 (5)	0.0027 (4)
C10	0.0230 (6)	0.0201 (6)	0.0190 (5)	−0.0108 (5)	0.0030 (5)	0.0034 (4)
C11	0.0209 (6)	0.0196 (5)	0.0152 (5)	−0.0091 (5)	0.0036 (4)	0.0010 (4)
C12	0.0181 (6)	0.0255 (6)	0.0128 (5)	−0.0096 (5)	0.0019 (4)	−0.0007 (4)
C13	0.0214 (6)	0.0187 (5)	0.0165 (5)	−0.0097 (5)	0.0011 (4)	0.0020 (4)
C14	0.0233 (6)	0.0206 (6)	0.0201 (6)	−0.0112 (5)	0.0011 (5)	0.0035 (5)

### Di-µ-chlorido-bis({(E)-5-(ethylamino)-4-methyl-2-[(pyridin-2-\ yl)diazenyl]phenolato}copper(II)) (3) Geometric parameters (Å, º)

**Table:**

Cu1—Cl^i^	2.6192 (4)	C4—C5	1.4436 (18)
Cu1—Cl	2.2985 (3)	C5—C6	1.4233 (17)
Cu1—O1	1.9654 (10)	C6—H6	0.96 (2)
Cu1—N1	1.9574 (11)	C7—C8	1.3954 (18)
Cu1—N3	1.9897 (11)	C8—H8	0.96 (2)
O1—C4	1.2974 (15)	C8—C9	1.3876 (19)
N1—N2	1.3008 (15)	C9—H9	0.92 (3)
N1—C5	1.3402 (16)	C9—C10	1.388 (2)
N2—C7	1.3963 (16)	C10—H10	0.94 (3)
N3—C7	1.3591 (17)	C10—C11	1.388 (2)
N3—C11	1.3385 (17)	C11—H11	0.92 (2)
N4—H4	0.77 (3)	C12—H12A	1.00 (3)
N4—C2	1.3490 (16)	C12—H12B	0.96 (3)
N4—C13	1.4539 (19)	C12—H12C	0.93 (2)
C1—C2	1.4567 (19)	C13—H13A	0.97 (2)
C1—C6	1.3647 (17)	C13—H13B	0.94 (2)
C1—C12	1.4967 (18)	C13—C14	1.5154 (19)
C2—C3	1.3989 (18)	C14—H14A	0.94 (2)
C3—H3	0.90 (2)	C14—H14B	0.97 (3)
C3—C4	1.4021 (17)	C14—H14C	0.96 (2)
			
Cl—Cu1—Cl^i^	90.307 (12)	C1—C6—C5	120.28 (12)
O1—Cu1—Cl^i^	93.88 (3)	C1—C6—H6	121.2 (13)
O1—Cu1—Cl	98.20 (3)	C5—C6—H6	118.5 (13)
O1—Cu1—N3	160.67 (4)	N3—C7—N2	118.82 (11)
N1—Cu1—Cl^i^	108.61 (3)	N3—C7—C8	121.30 (11)
N1—Cu1—Cl	161.00 (4)	C8—C7—N2	119.86 (12)
N1—Cu1—O1	82.69 (4)	C7—C8—H8	120.2 (14)
N1—Cu1—N3	77.99 (4)	C9—C8—C7	118.74 (13)
N3—Cu1—Cl	99.86 (3)	C9—C8—H8	121.0 (14)
N3—Cu1—Cl^i^	92.90 (4)	C8—C9—H9	116.4 (16)
Cu1—Cl—Cu1^i^	89.693 (12)	C8—C9—C10	119.56 (13)
C4—O1—Cu1	111.30 (8)	C10—C9—H9	124.0 (17)
N2—N1—Cu1	121.35 (8)	C9—C10—H10	122.3 (16)
N2—N1—C5	124.44 (11)	C9—C10—C11	118.88 (12)
C5—N1—Cu1	114.21 (9)	C11—C10—H10	118.7 (16)
N1—N2—C7	109.40 (10)	N3—C11—C10	122.01 (13)
C7—N3—Cu1	112.44 (8)	N3—C11—H11	113.9 (14)
C11—N3—Cu1	127.97 (10)	C10—C11—H11	124.0 (14)
C11—N3—C7	119.48 (12)	C1—C12—H12A	109.4 (14)
C2—N4—H4	118.0 (19)	C1—C12—H12B	111.7 (16)
C2—N4—C13	124.10 (12)	C1—C12—H12C	112.1 (15)
C13—N4—H4	117.8 (19)	H12A—C12—H12B	107 (2)
C2—C1—C12	119.00 (11)	H12A—C12—H12C	109 (2)
C6—C1—C2	118.99 (11)	H12B—C12—H12C	108 (2)
C6—C1—C12	121.99 (12)	N4—C13—H13A	112.4 (14)
N4—C2—C1	117.71 (11)	N4—C13—H13B	107.4 (14)
N4—C2—C3	121.30 (12)	N4—C13—C14	110.26 (12)
C3—C2—C1	120.99 (11)	H13A—C13—H13B	108.9 (19)
C2—C3—H3	121.3 (15)	C14—C13—H13A	108.5 (14)
C2—C3—C4	120.54 (12)	C14—C13—H13B	109.2 (14)
C4—C3—H3	118.2 (15)	C13—C14—H14A	109.6 (14)
O1—C4—C3	122.38 (12)	C13—C14—H14B	112.8 (16)
O1—C4—C5	119.72 (11)	C13—C14—H14C	112.1 (13)
C3—C4—C5	117.89 (11)	H14A—C14—H14B	109 (2)
N1—C5—C4	111.97 (11)	H14A—C14—H14C	104.7 (19)
N1—C5—C6	126.70 (12)	H14B—C14—H14C	108 (2)
C6—C5—C4	121.28 (11)		
			
Cu1—O1—C4—C3	175.56 (10)	C2—C1—C6—C5	2.1 (2)
Cu1—O1—C4—C5	−3.62 (15)	C2—C3—C4—O1	−179.58 (13)
Cu1—N1—N2—C7	0.45 (15)	C2—C3—C4—C5	−0.39 (19)
Cu1—N1—C5—C4	−0.01 (14)	C3—C4—C5—N1	−176.72 (12)
Cu1—N1—C5—C6	−177.55 (11)	C3—C4—C5—C6	0.98 (19)
Cu1—N3—C7—N2	0.24 (15)	C4—C5—C6—C1	−1.9 (2)
Cu1—N3—C7—C8	−178.40 (11)	C5—N1—N2—C7	−179.00 (12)
Cu1—N3—C11—C10	177.82 (12)	C6—C1—C2—N4	178.01 (12)
O1—C4—C5—N1	2.50 (18)	C6—C1—C2—C3	−1.5 (2)
O1—C4—C5—C6	−179.81 (12)	C7—N3—C11—C10	1.9 (2)
N1—N2—C7—N3	−0.44 (17)	C7—C8—C9—C10	−0.2 (2)
N1—N2—C7—C8	178.23 (12)	C8—C9—C10—C11	0.2 (2)
N1—C5—C6—C1	175.44 (13)	C9—C10—C11—N3	−1.1 (2)
N2—N1—C5—C4	179.48 (12)	C11—N3—C7—N2	176.77 (13)
N2—N1—C5—C6	1.9 (2)	C11—N3—C7—C8	−1.9 (2)
N2—C7—C8—C9	−177.61 (13)	C12—C1—C2—N4	−3.82 (19)
N3—C7—C8—C9	1.0 (2)	C12—C1—C2—C3	176.62 (13)
N4—C2—C3—C4	−178.88 (13)	C12—C1—C6—C5	−175.99 (13)
C1—C2—C3—C4	0.7 (2)	C13—N4—C2—C1	−179.80 (13)
C2—N4—C13—C14	172.24 (13)	C13—N4—C2—C3	−0.2 (2)

Symmetry code: (i) −*x*+1, −*y*+1, −*z*.

### Di-µ-chlorido-bis({(E)-5-(ethylamino)-4-methyl-2-[(pyridin-2-\ yl)diazenyl]phenolato}copper(II)) (3) Hydrogen-bond geometry (Å, º)

**Table:**

*D*—H···*A*	*D*—H	H···*A*	*D*···*A*	*D*—H···*A*
C9—H9···Cl^ii^	0.92 (3)	2.91 (3)	3.6514 (14)	138 (2)
C9—H9···O1^ii^	0.92 (3)	2.59 (3)	3.1592 (18)	120 (2)
C11—H11···Cl	0.92 (2)	2.93 (2)	3.4825 (15)	120.2 (17)
C11—H11···O1^i^	0.92 (2)	2.60 (2)	3.4083 (18)	146.5 (19)
C12—H12*B*···O1^iii^	0.96 (3)	2.85 (3)	3.7902 (19)	169 (2)
C12—H12*C*···Cl^iv^	0.93 (2)	3.00 (2)	3.4542 (15)	111.9 (17)
C14—H14*B*···Cl^v^	0.97 (3)	2.94 (3)	3.6821 (16)	134.8 (19)

Symmetry codes: (i) −*x*+1, −*y*+1, −*z*; (ii) *x*, *y*+1, *z*; (iii) −*x*, −*y*+1, −*z*+1; (iv) −*x*+1, −*y*+1, −*z*+1; (v) *x*−1, *y*, *z*+1.

### Chloridobis(1,10-phenanthroline)copper(II) chloride tetrahydrate (4) Crystal data

**Table:**

[CuCl(C_12_H_8_N_2_)_2_]Cl·4H_2_O	*F*(000) = 2328
*M**_r_* = 566.91	*D*_x_ = 1.485 Mg m^−^^3^
Monoclinic, *C*2/*c*	Mo *K*α radiation, λ = 0.71073 Å
*a* = 23.1874 (7) Å	Cell parameters from 9891 reflections
*b* = 30.2708 (9) Å	θ = 2.7–33.0°
*c* = 7.2839 (2) Å	µ = 1.11 mm^−^^1^
β = 97.235 (1)°	*T* = 100 K
*V* = 5071.9 (3) Å^3^	Plate, clear greenish green
*Z* = 8	0.2 × 0.1 × 0.05 mm

### Chloridobis(1,10-phenanthroline)copper(II) chloride tetrahydrate (4) Data collection

**Table:**

Bruker D8 Venture diffractometer	5840 reflections with *I* > 2σ(*I*)
θ and ω scans	*R*_int_ = 0.029
Absorption correction: multi-scan (SADABS; Krause et al., 2015)	θ_max_ = 28.3°, θ_min_ = 3.5°
*T*_min_ = 0.89, *T*_max_ = 0.95	*h* = −30→29
67930 measured reflections	*k* = −40→40
6173 independent reflections	*l* = −9→9

### Chloridobis(1,10-phenanthroline)copper(II) chloride tetrahydrate (4) Refinement

**Table:**

Refinement on *F*^2^	0 restraints
Least-squares matrix: full	Hydrogen site location: mixed
*R*[*F*^2^ > 2σ(*F*^2^)] = 0.045	H atoms treated by a mixture of independent and constrained refinement
*wR*(*F*^2^) = 0.130	*w* = 1/[σ^2^(*F*_o_^2^) + (0.0642*P*)^2^ + 16.2399*P*] where *P* = (*F*_o_^2^ + 2*F*_c_^2^)/3
*S* = 1.05	(Δ/σ)_max_ < 0.001
6173 reflections	Δρ_max_ = 1.06 e Å^−^^3^
364 parameters	Δρ_min_ = −0.48 e Å^−^^3^

### Chloridobis(1,10-phenanthroline)copper(II) chloride tetrahydrate (4) Special details

**Table:**

Geometry. All esds (except the esd in the dihedral angle between two l.s. planes) are estimated using the full covariance matrix. The cell esds are taken into account individually in the estimation of esds in distances, angles and torsion angles; correlations between esds in cell parameters are only used when they are defined by crystal symmetry. An approximate (isotropic) treatment of cell esds is used for estimating esds involving l.s. planes.

### Chloridobis(1,10-phenanthroline)copper(II) chloride tetrahydrate (4) Fractional atomic coordinates and isotropic or equivalent isotropic displacement parameters (Å^2^)

**Table:**

	*x*	*y*	*z*	*U*_iso_*/*U*_eq_	Occ. (<1)
Cu1	0.27540 (2)	0.11858 (2)	0.34073 (4)	0.02177 (10)	
Cl1	0.29111 (3)	0.14735 (2)	0.05112 (7)	0.02655 (13)	
N1	0.22658 (8)	0.15474 (6)	0.5111 (3)	0.0219 (4)	
N2	0.33904 (9)	0.15480 (6)	0.4699 (3)	0.0249 (4)	
N3	0.20905 (8)	0.08317 (6)	0.2241 (3)	0.0237 (4)	
N4	0.31053 (8)	0.05547 (6)	0.3980 (2)	0.0215 (3)	
C2	0.11535 (11)	0.07030 (9)	0.0549 (4)	0.0339 (5)	
H2	0.080421	0.082186	−0.008153	0.041*	
C3	0.12262 (11)	0.02558 (9)	0.0713 (3)	0.0320 (5)	
H3	0.092669	0.006179	0.019772	0.038*	
C4	0.17442 (10)	0.00847 (8)	0.1643 (3)	0.0262 (4)	
C5	0.21693 (10)	0.03886 (7)	0.2389 (3)	0.0223 (4)	
C6	0.18679 (12)	−0.03787 (8)	0.1861 (3)	0.0294 (5)	
H6	0.158392	−0.058845	0.137680	0.035*	
C7	0.23830 (12)	−0.05215 (8)	0.2746 (3)	0.0299 (5)	
H7	0.245351	−0.082976	0.287392	0.036*	
C8	0.28242 (11)	−0.02157 (7)	0.3493 (3)	0.0248 (4)	
C9	0.27151 (9)	0.02397 (7)	0.3316 (3)	0.0215 (4)	
C10	0.33706 (11)	−0.03444 (8)	0.4395 (3)	0.0288 (5)	
H10	0.346589	−0.064863	0.454168	0.035*	
C11	0.37645 (11)	−0.00274 (8)	0.5063 (3)	0.0288 (5)	
H11	0.413550	−0.010993	0.567009	0.035*	
C12	0.36138 (10)	0.04220 (8)	0.4839 (3)	0.0253 (4)	
H12	0.388785	0.063878	0.532360	0.030*	
C13	0.17066 (10)	0.15345 (7)	0.5335 (3)	0.0254 (4)	
H13	0.147100	0.130709	0.473677	0.030*	
C14	0.14494 (11)	0.18425 (8)	0.6418 (3)	0.0302 (5)	
H14	0.104956	0.181807	0.656823	0.036*	
C15	0.17792 (12)	0.21793 (8)	0.7258 (3)	0.0311 (5)	
H15	0.160782	0.239449	0.796876	0.037*	
C16	0.23741 (11)	0.22029 (7)	0.7056 (3)	0.0265 (5)	
C17	0.25979 (10)	0.18725 (7)	0.5984 (3)	0.0223 (4)	
C18	0.32006 (10)	0.18741 (7)	0.5757 (3)	0.0237 (4)	
C19	0.35695 (11)	0.22065 (8)	0.6583 (3)	0.0289 (5)	
C20	0.33264 (12)	0.25421 (8)	0.7643 (3)	0.0329 (5)	
H20	0.357096	0.276952	0.820068	0.040*	
C21	0.27566 (12)	0.25416 (8)	0.7864 (3)	0.0319 (5)	
H21	0.260710	0.276953	0.856655	0.038*	
C22	0.41563 (12)	0.21906 (9)	0.6275 (4)	0.0359 (6)	
H22	0.442180	0.240951	0.679455	0.043*	
C23	0.43414 (12)	0.18584 (10)	0.5223 (4)	0.0381 (6)	
H23	0.473811	0.184258	0.502541	0.046*	
C24	0.39459 (11)	0.15413 (9)	0.4438 (4)	0.0313 (5)	
H24	0.407952	0.131427	0.369617	0.038*	
C27	0.15990 (10)	0.09837 (8)	0.1320 (3)	0.0297 (5)	
H27	0.154850	0.129383	0.118071	0.036*	
Cl2	0.46154 (12)	0.28339 (11)	0.0469 (4)	0.0402 (6)	0.25
Cl3	0.500000	0.3448 (2)	0.750000	0.0671 (14)	0.25
Cl4	0.45206 (18)	0.41920 (18)	0.5363 (8)	0.1083 (15)	0.35
Cl5	0.500000	0.0804 (2)	0.250000	0.0353 (12)*	0.15
Cl6	0.4700 (2)	0.33053 (15)	0.8319 (8)	0.0601 (11)	0.2
O1	0.42362 (11)	0.17008 (13)	0.0286 (4)	0.0799 (10)	
H1A	0.387673	0.166966	0.029787	0.087 (16)*	
H1B	0.442713	0.147816	0.083907	0.131*	
O2	0.45025 (16)	0.25165 (18)	0.1188 (6)	0.0775 (13)	0.75
O3	0.500000	0.37225 (18)	0.250000	0.0705 (14)	0.8
O4	0.5355 (7)	0.3708 (6)	0.890 (2)	0.051 (4)*	0.15
O5	0.4383 (4)	0.3247 (3)	0.9597 (13)	0.062 (2)*	0.3
O6	0.500000	0.4802 (5)	0.750000	0.078 (6)	0.3
O7	0.500000	0.5117 (8)	0.750000	0.143 (8)	0.5
O8	0.500000	0.0553 (2)	0.250000	0.073 (3)	0.5
O9	0.4821 (6)	0.0907 (4)	0.0297 (18)	0.057 (3)*	0.2
O10	0.5421 (3)	0.0980 (3)	0.6321 (11)	0.0487 (17)*	0.3
O11	0.4997 (5)	0.0460 (3)	0.6952 (12)	0.069 (3)*	0.3

### Chloridobis(1,10-phenanthroline)copper(II) chloride tetrahydrate (4) Atomic displacement parameters (Å^2^)

**Table:**

	*U*^11^	*U*^22^	*U*^33^	*U*^12^	*U*^13^	*U*^23^
Cu1	0.02256 (16)	0.01895 (15)	0.02420 (16)	0.00082 (9)	0.00442 (10)	−0.00141 (9)
Cl1	0.0350 (3)	0.0203 (2)	0.0253 (3)	0.00274 (19)	0.0076 (2)	0.00302 (18)
N1	0.0266 (9)	0.0171 (8)	0.0227 (8)	0.0038 (7)	0.0052 (7)	0.0018 (6)
N2	0.0261 (9)	0.0222 (9)	0.0266 (9)	0.0002 (7)	0.0038 (7)	0.0006 (7)
N3	0.0249 (9)	0.0235 (9)	0.0233 (9)	0.0011 (7)	0.0055 (7)	−0.0010 (7)
N4	0.0252 (9)	0.0209 (8)	0.0195 (8)	0.0024 (7)	0.0071 (7)	0.0006 (7)
C2	0.0250 (11)	0.0440 (14)	0.0327 (12)	0.0025 (10)	0.0028 (9)	−0.0055 (11)
C3	0.0281 (11)	0.0416 (13)	0.0273 (11)	−0.0072 (10)	0.0073 (9)	−0.0083 (10)
C4	0.0307 (11)	0.0300 (11)	0.0199 (10)	−0.0055 (9)	0.0114 (8)	−0.0042 (8)
C5	0.0268 (10)	0.0236 (10)	0.0182 (9)	−0.0014 (8)	0.0094 (8)	−0.0012 (7)
C6	0.0405 (13)	0.0265 (11)	0.0234 (10)	−0.0101 (9)	0.0131 (9)	−0.0054 (8)
C7	0.0476 (14)	0.0211 (10)	0.0240 (11)	−0.0051 (9)	0.0159 (10)	−0.0020 (8)
C8	0.0372 (12)	0.0204 (10)	0.0194 (10)	0.0008 (8)	0.0130 (9)	0.0010 (7)
C9	0.0287 (10)	0.0205 (10)	0.0171 (9)	−0.0005 (8)	0.0100 (8)	−0.0002 (7)
C10	0.0414 (13)	0.0217 (10)	0.0257 (11)	0.0069 (9)	0.0134 (9)	0.0047 (8)
C11	0.0329 (12)	0.0289 (11)	0.0258 (11)	0.0088 (9)	0.0085 (9)	0.0054 (9)
C12	0.0265 (10)	0.0266 (11)	0.0235 (10)	0.0035 (8)	0.0066 (8)	0.0013 (8)
C13	0.0272 (11)	0.0222 (10)	0.0278 (11)	0.0043 (8)	0.0070 (8)	0.0030 (8)
C14	0.0306 (11)	0.0323 (12)	0.0288 (11)	0.0111 (9)	0.0079 (9)	0.0033 (9)
C15	0.0404 (13)	0.0295 (11)	0.0232 (10)	0.0167 (10)	0.0038 (9)	0.0000 (9)
C16	0.0381 (12)	0.0203 (10)	0.0196 (9)	0.0092 (9)	−0.0016 (9)	0.0024 (8)
C17	0.0293 (11)	0.0177 (9)	0.0194 (9)	0.0047 (8)	0.0008 (8)	0.0029 (7)
C18	0.0294 (11)	0.0188 (9)	0.0220 (10)	0.0024 (8)	0.0000 (8)	0.0036 (8)
C19	0.0351 (12)	0.0216 (10)	0.0275 (11)	0.0000 (9)	−0.0060 (9)	0.0041 (8)
C20	0.0461 (14)	0.0206 (10)	0.0280 (11)	0.0020 (9)	−0.0113 (10)	−0.0005 (9)
C21	0.0495 (15)	0.0198 (10)	0.0236 (10)	0.0101 (10)	−0.0065 (10)	−0.0020 (8)
C22	0.0333 (12)	0.0306 (12)	0.0407 (14)	−0.0069 (10)	−0.0069 (10)	0.0035 (10)
C23	0.0258 (12)	0.0409 (14)	0.0468 (15)	−0.0038 (10)	0.0019 (10)	0.0046 (12)
C24	0.0266 (11)	0.0315 (12)	0.0365 (13)	−0.0001 (9)	0.0065 (9)	0.0012 (10)
C27	0.0267 (11)	0.0313 (12)	0.0310 (12)	0.0043 (9)	0.0035 (9)	−0.0020 (9)
Cl2	0.0320 (12)	0.0532 (16)	0.0341 (12)	−0.0030 (11)	−0.0004 (10)	−0.0101 (12)
Cl3	0.080 (4)	0.062 (3)	0.056 (3)	0.000	−0.006 (3)	0.000
Cl4	0.072 (2)	0.118 (3)	0.141 (4)	0.034 (2)	0.037 (2)	−0.002 (3)
Cl6	0.057 (2)	0.042 (2)	0.081 (3)	0.0021 (18)	0.008 (2)	0.001 (2)
O1	0.0370 (13)	0.150 (3)	0.0549 (15)	−0.0042 (16)	0.0139 (11)	0.0370 (18)
O2	0.0407 (18)	0.115 (4)	0.074 (3)	0.020 (2)	−0.0037 (17)	−0.031 (3)
O3	0.069 (3)	0.065 (3)	0.072 (3)	0.000	−0.011 (3)	0.000
O6	0.034 (7)	0.077 (9)	0.132 (17)	0.000	0.050 (8)	0.000
O7	0.029 (4)	0.37 (3)	0.034 (4)	0.000	0.006 (3)	0.000
O8	0.019 (3)	0.046 (4)	0.141 (8)	0.000	−0.043 (4)	0.000

### Chloridobis(1,10-phenanthroline)copper(II) chloride tetrahydrate (4) Geometric parameters (Å, º)

**Table:**

Cu1—Cl1	2.3527 (6)	C11—H11	0.9500
Cu1—N1	2.0914 (18)	C11—C12	1.409 (3)
Cu1—N2	1.979 (2)	C12—H12	0.9500
Cu1—N3	1.977 (2)	C13—H13	0.9500
Cu1—N4	2.0979 (18)	C13—C14	1.402 (3)
N1—C13	1.328 (3)	C14—H14	0.9500
N1—C17	1.357 (3)	C14—C15	1.371 (4)
N2—C18	1.359 (3)	C15—H15	0.9500
N2—C24	1.326 (3)	C15—C16	1.407 (4)
N3—C5	1.356 (3)	C16—C17	1.407 (3)
N3—C27	1.330 (3)	C16—C21	1.433 (4)
N4—C9	1.361 (3)	C17—C18	1.428 (3)
N4—C12	1.326 (3)	C18—C19	1.406 (3)
C2—H2	0.9500	C19—C20	1.434 (4)
C2—C3	1.367 (4)	C19—C22	1.408 (4)
C2—C27	1.400 (4)	C20—H20	0.9500
C3—H3	0.9500	C20—C21	1.351 (4)
C3—C4	1.402 (4)	C21—H21	0.9500
C4—C5	1.407 (3)	C22—H22	0.9500
C4—C6	1.437 (3)	C22—C23	1.366 (4)
C5—C9	1.430 (3)	C23—H23	0.9500
C6—H6	0.9500	C23—C24	1.399 (4)
C6—C7	1.354 (4)	C24—H24	0.9500
C7—H7	0.9500	C27—H27	0.9500
C7—C8	1.435 (3)	Cl3—Cl6	1.063 (6)
C8—C9	1.405 (3)	O1—H1A	0.8400
C8—C10	1.407 (4)	O1—H1B	0.8759
C10—H10	0.9500	O11—O11^i^	0.797 (18)
C10—C11	1.371 (4)		
			
N1—Cu1—Cl1	119.65 (5)	C11—C10—H10	120.2
N1—Cu1—N4	125.75 (7)	C10—C11—H11	120.3
N2—Cu1—Cl1	91.76 (6)	C10—C11—C12	119.4 (2)
N2—Cu1—N1	81.65 (8)	C12—C11—H11	120.3
N2—Cu1—N4	99.35 (8)	N4—C12—C11	122.7 (2)
N3—Cu1—Cl1	90.89 (6)	N4—C12—H12	118.7
N3—Cu1—N1	95.07 (8)	C11—C12—H12	118.7
N3—Cu1—N2	176.52 (8)	N1—C13—H13	118.6
N3—Cu1—N4	81.58 (8)	N1—C13—C14	122.8 (2)
N4—Cu1—Cl1	114.55 (5)	C14—C13—H13	118.6
C13—N1—Cu1	131.88 (16)	C13—C14—H14	120.3
C13—N1—C17	117.96 (19)	C15—C14—C13	119.5 (2)
C17—N1—Cu1	109.88 (14)	C15—C14—H14	120.3
C18—N2—Cu1	113.38 (15)	C14—C15—H15	120.3
C24—N2—Cu1	127.10 (17)	C14—C15—C16	119.3 (2)
C24—N2—C18	118.8 (2)	C16—C15—H15	120.3
C5—N3—Cu1	114.35 (15)	C15—C16—C17	117.2 (2)
C27—N3—Cu1	126.91 (17)	C15—C16—C21	123.9 (2)
C27—N3—C5	118.7 (2)	C17—C16—C21	119.0 (2)
C9—N4—Cu1	110.08 (14)	N1—C17—C16	123.2 (2)
C12—N4—Cu1	132.06 (16)	N1—C17—C18	116.99 (19)
C12—N4—C9	117.87 (19)	C16—C17—C18	119.8 (2)
C3—C2—H2	120.4	N2—C18—C17	117.1 (2)
C3—C2—C27	119.3 (2)	N2—C18—C19	122.6 (2)
C27—C2—H2	120.4	C19—C18—C17	120.3 (2)
C2—C3—H3	120.1	C18—C19—C20	118.6 (2)
C2—C3—C4	119.8 (2)	C18—C19—C22	117.1 (2)
C4—C3—H3	120.1	C22—C19—C20	124.3 (2)
C3—C4—C5	117.5 (2)	C19—C20—H20	119.3
C3—C4—C6	124.2 (2)	C21—C20—C19	121.3 (2)
C5—C4—C6	118.4 (2)	C21—C20—H20	119.3
N3—C5—C4	122.4 (2)	C16—C21—H21	119.5
N3—C5—C9	116.8 (2)	C20—C21—C16	121.0 (2)
C4—C5—C9	120.8 (2)	C20—C21—H21	119.5
C4—C6—H6	119.5	C19—C22—H22	120.2
C7—C6—C4	121.1 (2)	C23—C22—C19	119.5 (2)
C7—C6—H6	119.5	C23—C22—H22	120.2
C6—C7—H7	119.4	C22—C23—H23	120.1
C6—C7—C8	121.2 (2)	C22—C23—C24	119.9 (3)
C8—C7—H7	119.4	C24—C23—H23	120.1
C9—C8—C7	119.1 (2)	N2—C24—C23	122.0 (2)
C9—C8—C10	117.1 (2)	N2—C24—H24	119.0
C10—C8—C7	123.8 (2)	C23—C24—H24	119.0
N4—C9—C5	117.14 (19)	N3—C27—C2	122.3 (2)
N4—C9—C8	123.5 (2)	N3—C27—H27	118.8
C8—C9—C5	119.4 (2)	C2—C27—H27	118.8
C8—C10—H10	120.2	H1A—O1—H1B	110.7
C11—C10—C8	119.5 (2)		
			
Cu1—N1—C13—C14	−172.78 (17)	C8—C10—C11—C12	0.3 (3)
Cu1—N1—C17—C16	172.37 (17)	C9—N4—C12—C11	0.9 (3)
Cu1—N1—C17—C18	−6.7 (2)	C9—C8—C10—C11	0.4 (3)
Cu1—N2—C18—C17	8.1 (2)	C10—C8—C9—N4	−0.5 (3)
Cu1—N2—C18—C19	−170.92 (17)	C10—C8—C9—C5	179.20 (19)
Cu1—N2—C24—C23	170.0 (2)	C10—C11—C12—N4	−1.0 (3)
Cu1—N3—C5—C4	−179.97 (16)	C12—N4—C9—C5	−179.83 (18)
Cu1—N3—C5—C9	−1.2 (2)	C12—N4—C9—C8	−0.1 (3)
Cu1—N3—C27—C2	−179.82 (18)	C13—N1—C17—C16	−2.3 (3)
Cu1—N4—C9—C5	0.5 (2)	C13—N1—C17—C18	178.66 (19)
Cu1—N4—C9—C8	−179.83 (16)	C13—C14—C15—C16	−1.6 (3)
Cu1—N4—C12—C11	−179.50 (16)	C14—C15—C16—C17	0.0 (3)
N1—C13—C14—C15	1.5 (4)	C14—C15—C16—C21	179.6 (2)
N1—C17—C18—N2	−0.6 (3)	C15—C16—C17—N1	2.1 (3)
N1—C17—C18—C19	178.50 (19)	C15—C16—C17—C18	−178.9 (2)
N2—C18—C19—C20	178.7 (2)	C15—C16—C21—C20	179.0 (2)
N2—C18—C19—C22	−0.1 (3)	C16—C17—C18—N2	−179.67 (19)
N3—C5—C9—N4	0.5 (3)	C16—C17—C18—C19	−0.6 (3)
N3—C5—C9—C8	−179.28 (18)	C17—N1—C13—C14	0.4 (3)
C2—C3—C4—C5	0.5 (3)	C17—C16—C21—C20	−1.4 (3)
C2—C3—C4—C6	−178.8 (2)	C17—C18—C19—C20	−0.3 (3)
C3—C2—C27—N3	−1.2 (4)	C17—C18—C19—C22	−179.0 (2)
C3—C4—C5—N3	0.3 (3)	C18—N2—C24—C23	0.2 (4)
C3—C4—C5—C9	−178.41 (19)	C18—C19—C20—C21	0.4 (3)
C3—C4—C6—C7	178.7 (2)	C18—C19—C22—C23	−0.6 (4)
C4—C5—C9—N4	179.26 (18)	C19—C20—C21—C16	0.4 (4)
C4—C5—C9—C8	−0.5 (3)	C19—C22—C23—C24	1.0 (4)
C4—C6—C7—C8	−0.2 (3)	C20—C19—C22—C23	−179.2 (2)
C5—N3—C27—C2	2.0 (3)	C21—C16—C17—N1	−177.6 (2)
C5—C4—C6—C7	−0.7 (3)	C21—C16—C17—C18	1.5 (3)
C6—C4—C5—N3	179.7 (2)	C22—C19—C20—C21	179.0 (2)
C6—C4—C5—C9	1.0 (3)	C22—C23—C24—N2	−0.8 (4)
C6—C7—C8—C9	0.7 (3)	C24—N2—C18—C17	179.3 (2)
C6—C7—C8—C10	−178.8 (2)	C24—N2—C18—C19	0.2 (3)
C7—C8—C9—N4	179.92 (19)	C27—N3—C5—C4	−1.6 (3)
C7—C8—C9—C5	−0.4 (3)	C27—N3—C5—C9	177.2 (2)
C7—C8—C10—C11	179.9 (2)	C27—C2—C3—C4	−0.2 (4)

Symmetry code: (i) −*x*+1, *y*, −*z*+3/2.

### Chloridobis(1,10-phenanthroline)copper(II) chloride tetrahydrate (4) Hydrogen-bond geometry (Å, º)

**Table:**

*D*—H···*A*	*D*—H	H···*A*	*D*···*A*	*D*—H···*A*
C11—H11···O11	0.95	2.72	3.354 (11)	125
C12—H12···O11	0.95	2.75	3.380 (12)	125
C13—H13···O4^ii^	0.95	2.58	3.261 (17)	129
C23—H23···O1^iii^	0.95	2.46	3.399 (4)	172
C27—H27···O5^iv^	0.95	2.57	3.267 (10)	130
O1—H1*A*···Cl1	0.84	2.34	3.173 (3)	171
O1—H1*B*···O9	0.88	2.02	2.758 (14)	142

Symmetry codes: (ii) *x*−1/2, −*y*+1/2, *z*−1/2; (iii) −*x*+1, *y*, −*z*+1/2; (iv) −*x*+1/2, −*y*+1/2, −*z*+1.
