# Supplementary material for: Crystal structures of di-μ-chlorido-bis­({(E)-5-(ethyl­amino)-4-methyl-2-[(pyridin-2-yl)diazen­yl]phen­o­lato}copper(II)) and chlorido­bis­(1,10-phen­anthroline)copper(II) chloride tetra­hydrate
Source: Acta Crystallogr E Crystallogr Commun. 2023 Feb 21;79(Pt 3):201–6. doi: 10.1107/S205698902300138X (PMC9993926; doi:10.1107/S205698902300138X)
Supplement: Supplementary file 4 [file e-79-00201-sup4.docx]

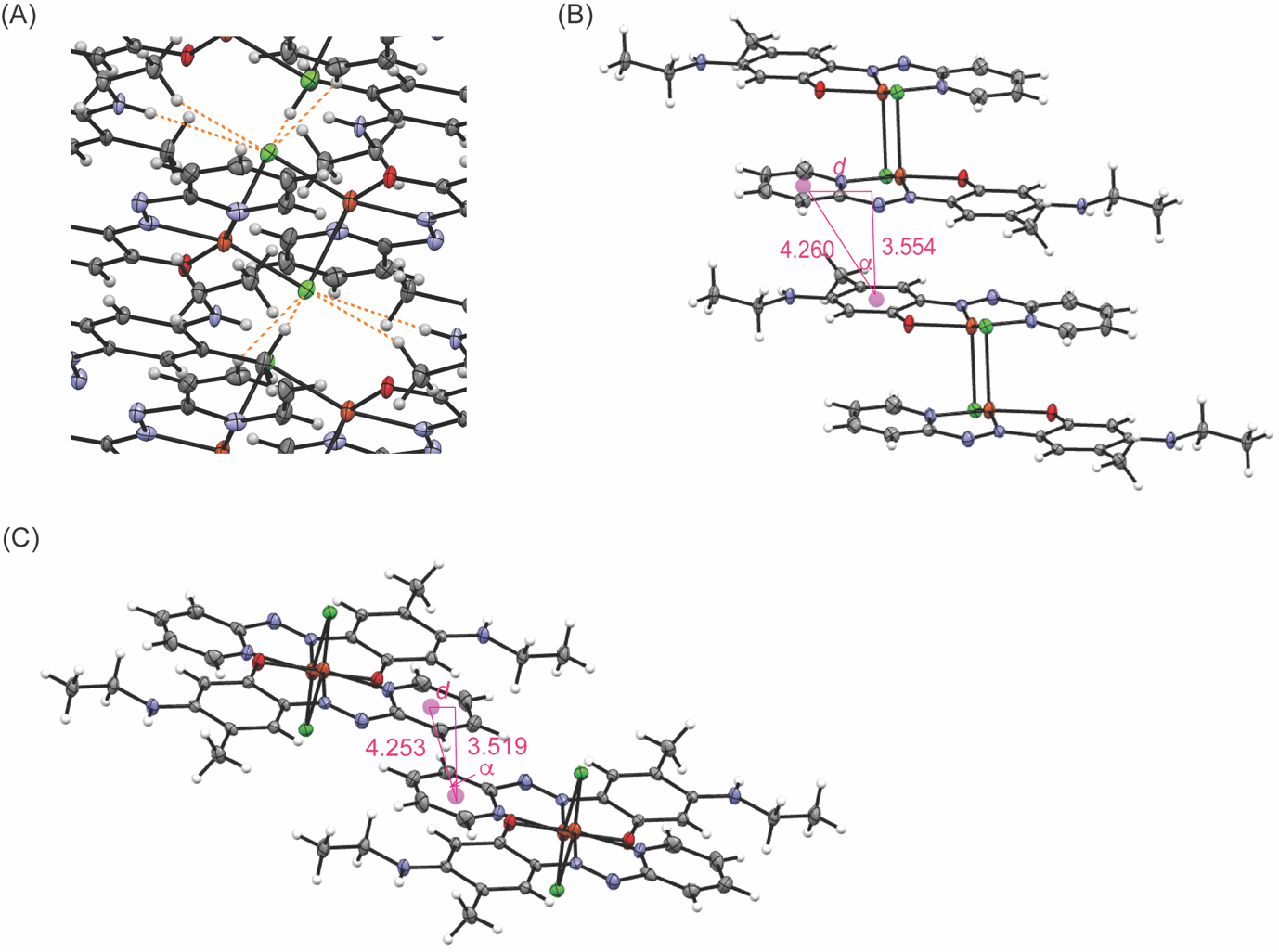


1. Intermolecular interactions in the crystal of **3** (see Tables 1 and 2 for numerical information). (A) Intermolecular hydrogen bonding interactions are shown in orange dashed lines: involving Cl (green ellipsoids) as an acceptor. The offset parallel π-π stacking interactions between the phenyl and the pyridine rings (B), and between the two pyridine rings (C). α is the angle between the ring normal and the vector between the ring centroids; *d* is the offset between the two aromatic rings.


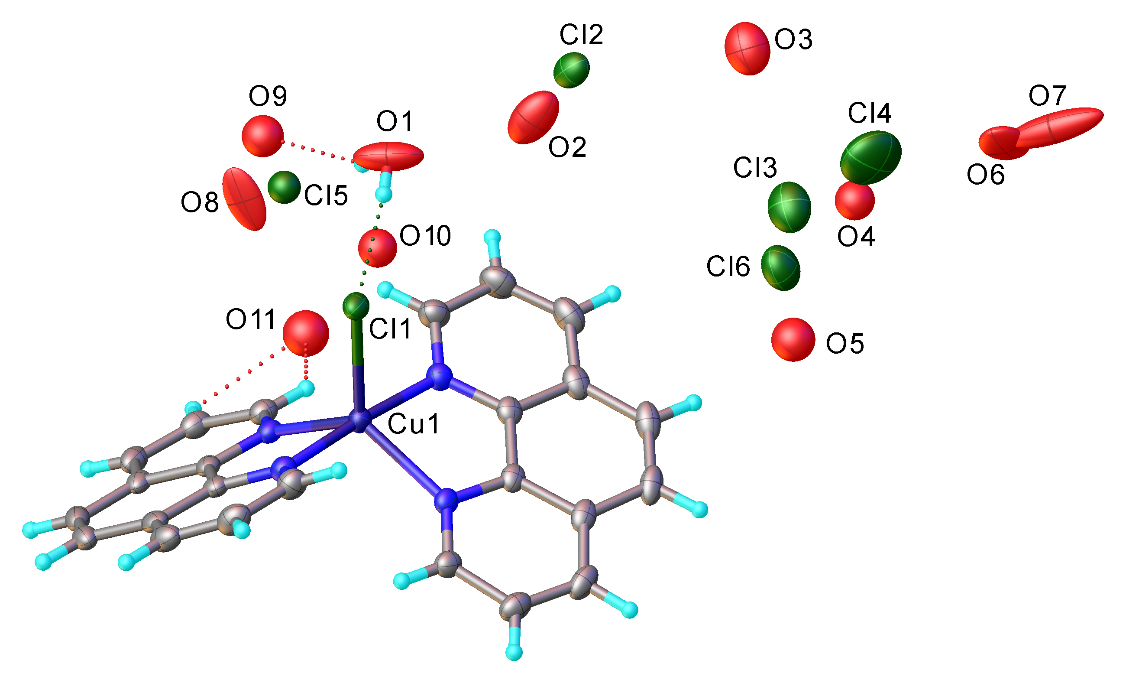


1. The asymmetric unit of **4** showing the disordered atoms


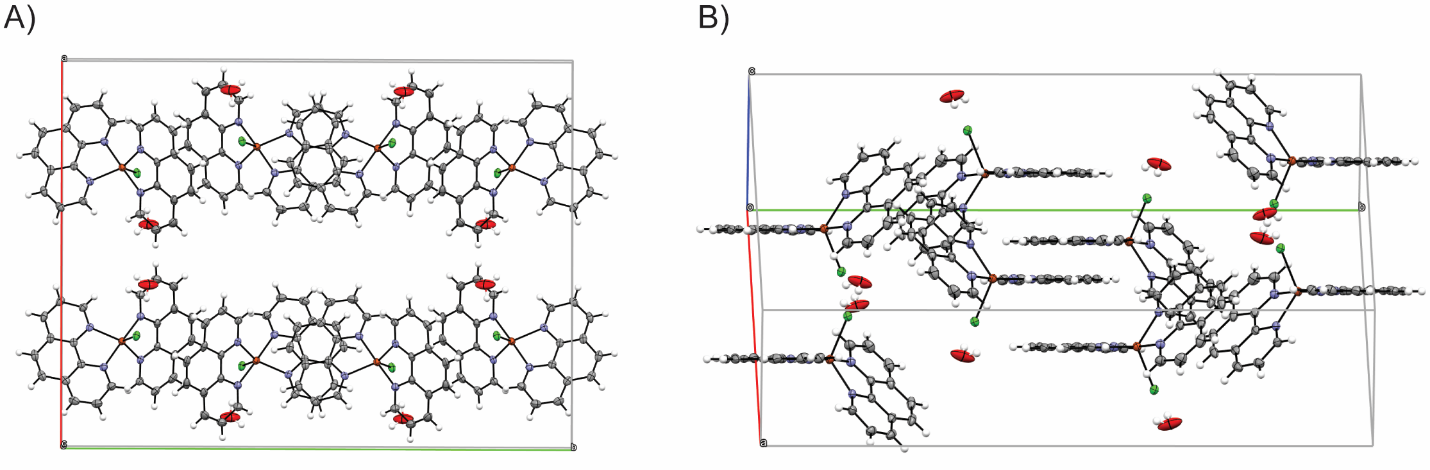


1. Crystal packing of **4** in different aspects. Disordered atoms are omitted.
